# Signs of Nature in Spine Radiology

**DOI:** 10.7759/cureus.2456

**Published:** 2018-04-10

**Authors:** MN Baig, Fergus Byrne, A Devitt, J P McCabe

**Affiliations:** 1 Trauma & Orthopaedics, Galway University Hospital

**Keywords:** spine, ivory vertebrae, scalloping vertebrae

## Abstract

As medical science developed over time, we have relied on natural imagery to help us recognise and remember things. In this review article, we will be discussing some radiological signs named because of their resemblance to the occurrences in the natural world.

## Introduction and background

Relating things to certain natural objects or events to remember is human nature. We have adapted to remember and recall information we can associate with our surroundings. Clinical and radiological signs are named after natural signs. In orthopaedics, many radiological findings are named for the natural phenomena of which they mimic.

## Review

We will discuss radiological signs in orthopaedics whose names are based on phenomena or observations in the natural world.

Butterfly vertebrae

A butterfly vertebra is a rare congenital symmetric fusion defect in the sagittal cleft vertebrae. It is caused by persistence of the notochord during the development of normal vertebrae, usually occurring during the third to sixth week of gestation when two lateral centers of chondrification fail to fuse together [1].

**Figure 1:**
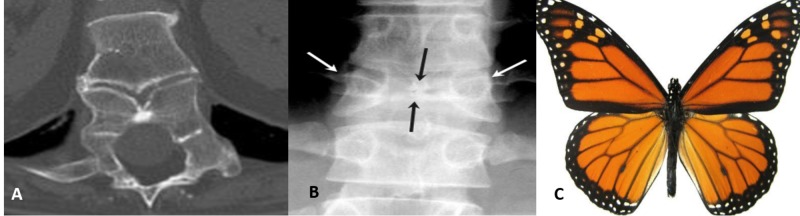
Butterfly vertebrae X-ray and computed tomography scan showing butterfly vertebrae.

It is mostly an incidental finding and rarely causes back pain. It can be easily confused with a compression fracture. There is a widening of the lateral parts of vertebrae, and a bony bridge may or may not form between two lateral fragments (Figure 1).

Scottie dog sign

Scottie dog sign is present in normal lumbar spine vertebrae present in an oblique view. The posterior elements of the vertebrae form the impression of a Scottie dog. The nose of the dog is the transverse process, the eye is the pedicle, the neck is the pars interarticularis, the front leg is the inferior articular facet, and the ear is the superior articular facet [2].

**Figure 2:**
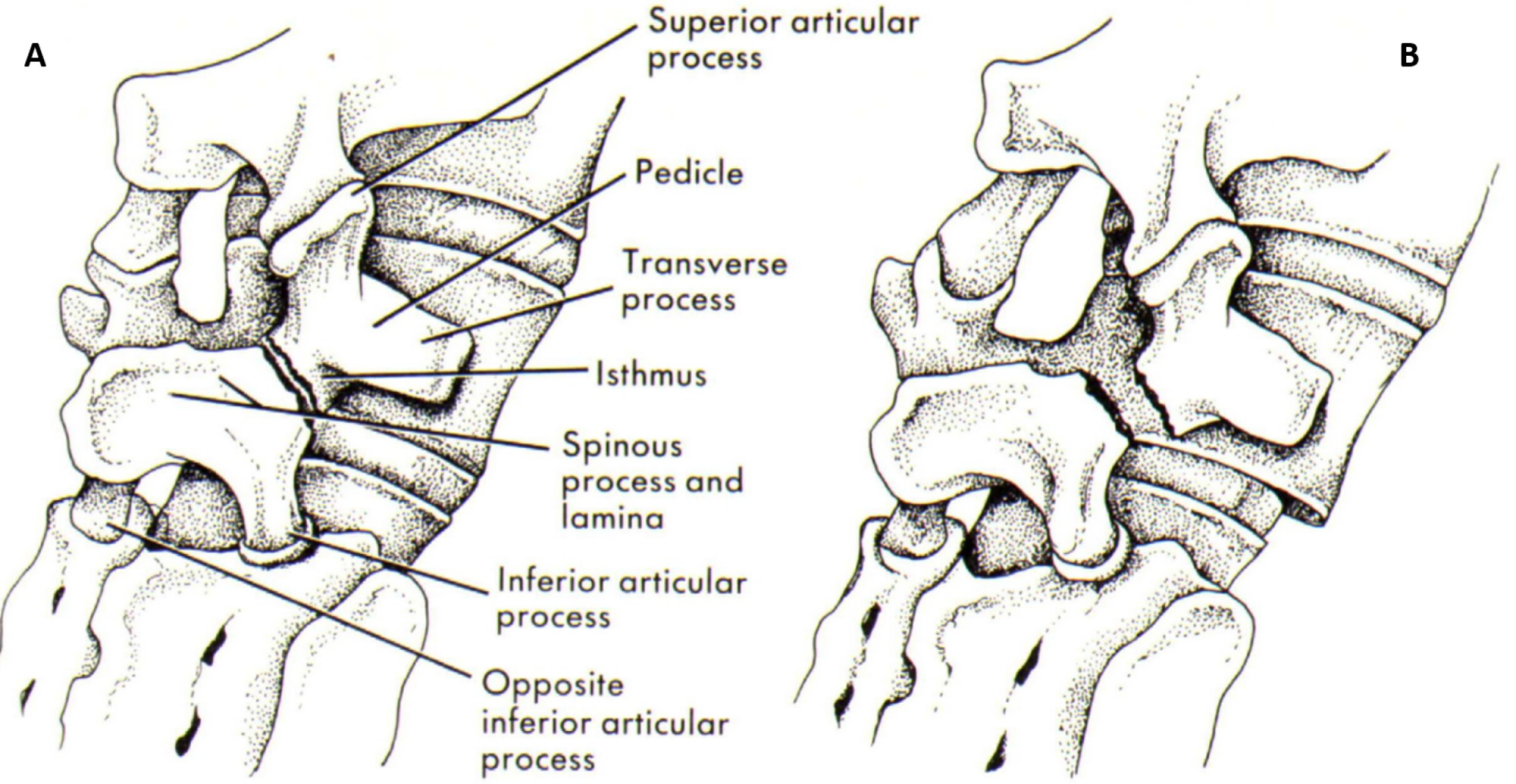
Scottie dog and pars interarticularis Illustration showing the resemblance to Scottie dog.

**Figure 3:**
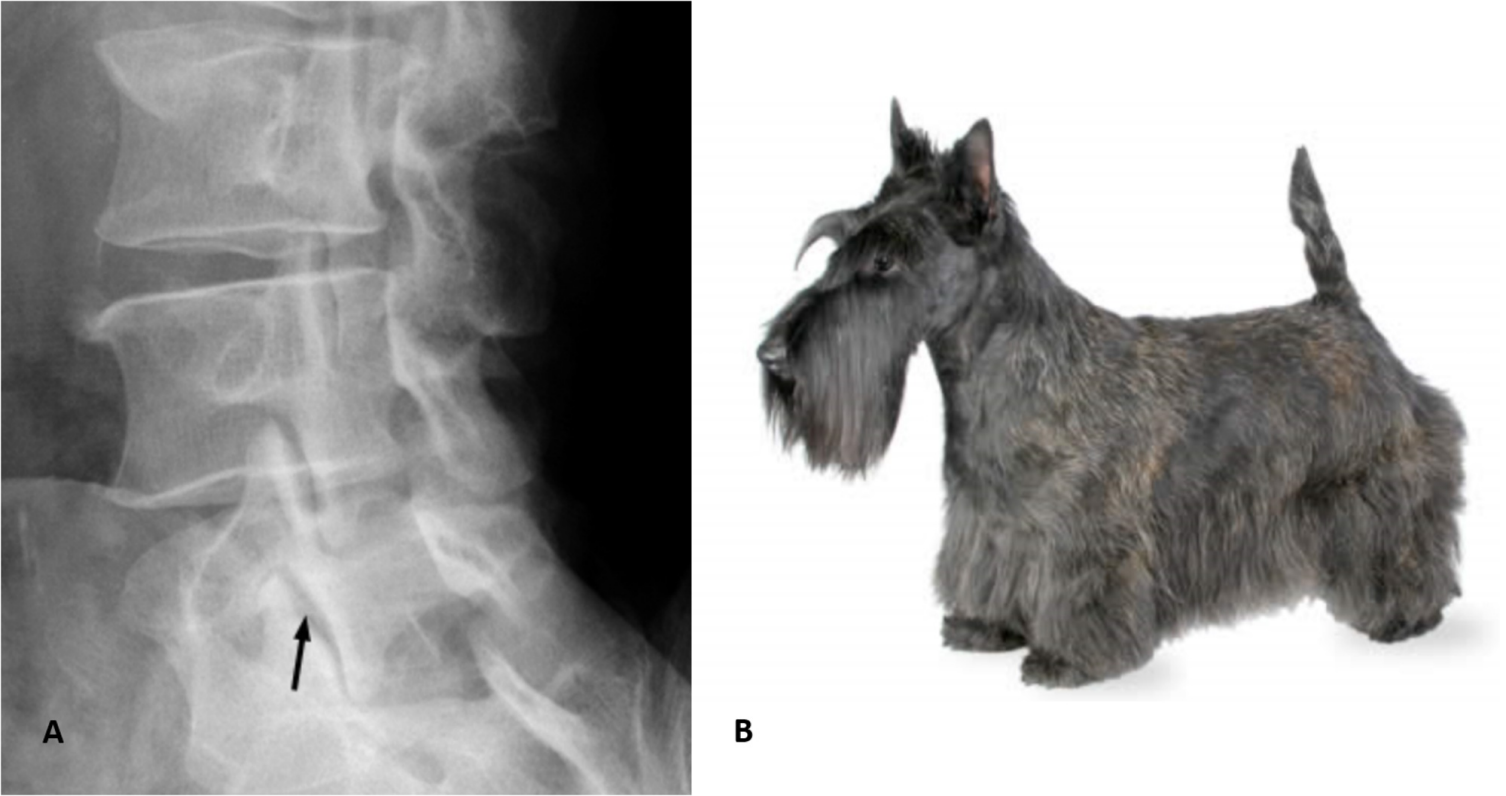
Scottie dog x-ray Scottie dog and x-ray showing resemblance.

The Scottie dog sign is very useful in the diagnosis of spondylosis where a pars interarticularis defect manifests itself as a defect of the neck (Figures 2-3).

Bamboo sign

The bamboo spine is a term used for the x-ray image of a spine affected by ankylosing spondylitis where the spine is affected by bridging syndesmophyte and sacroiliitis. There is enthesis formation between individual adjoining vertebrae. The outer fibres of the annulus fibrosis of the intervertebral discs ossify to form syndesmophytes bridges between the vertebrae. It is evident on an anteroposterior x-ray of the spine [3].

**Figure 4:**
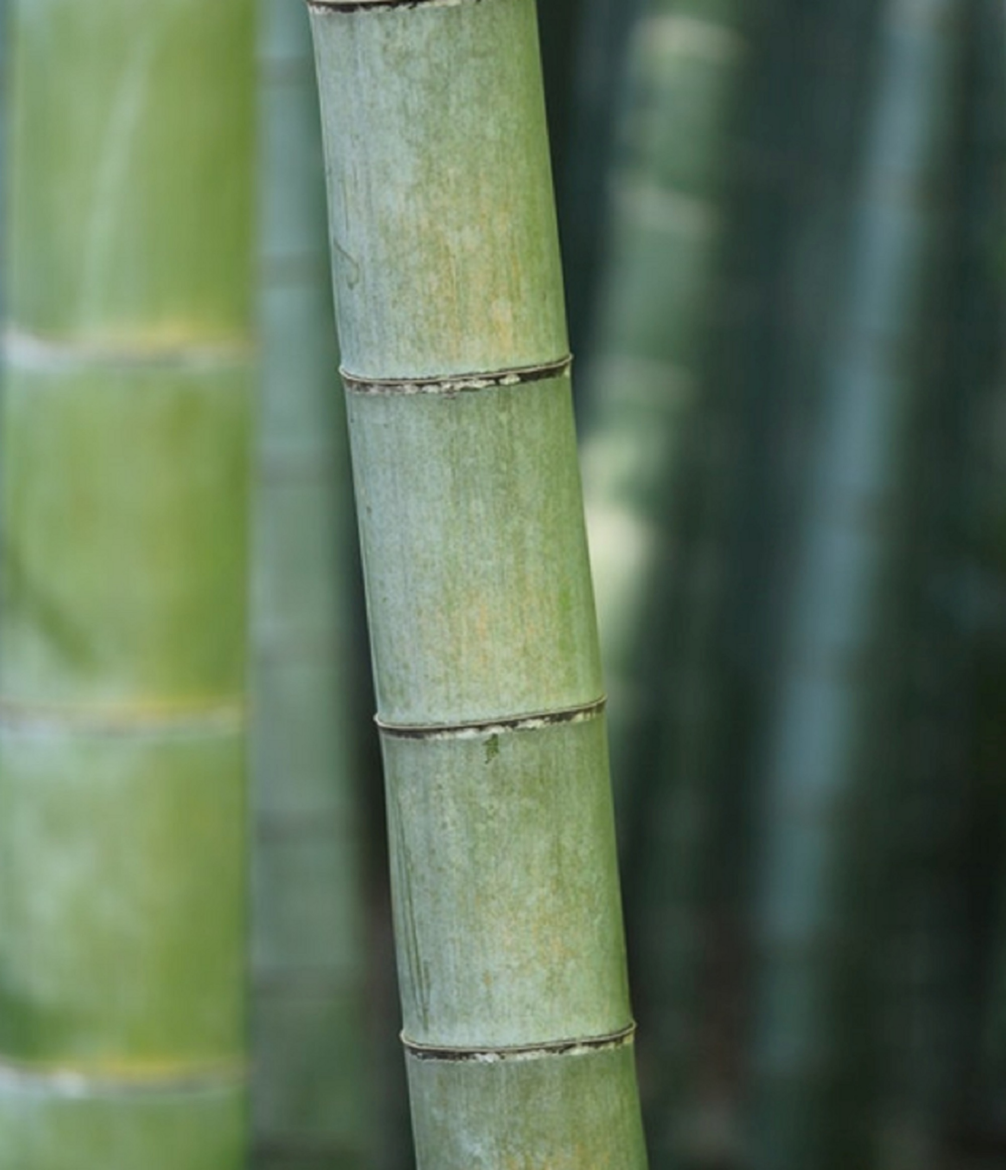
Bamboo plant

**Figure 5:**
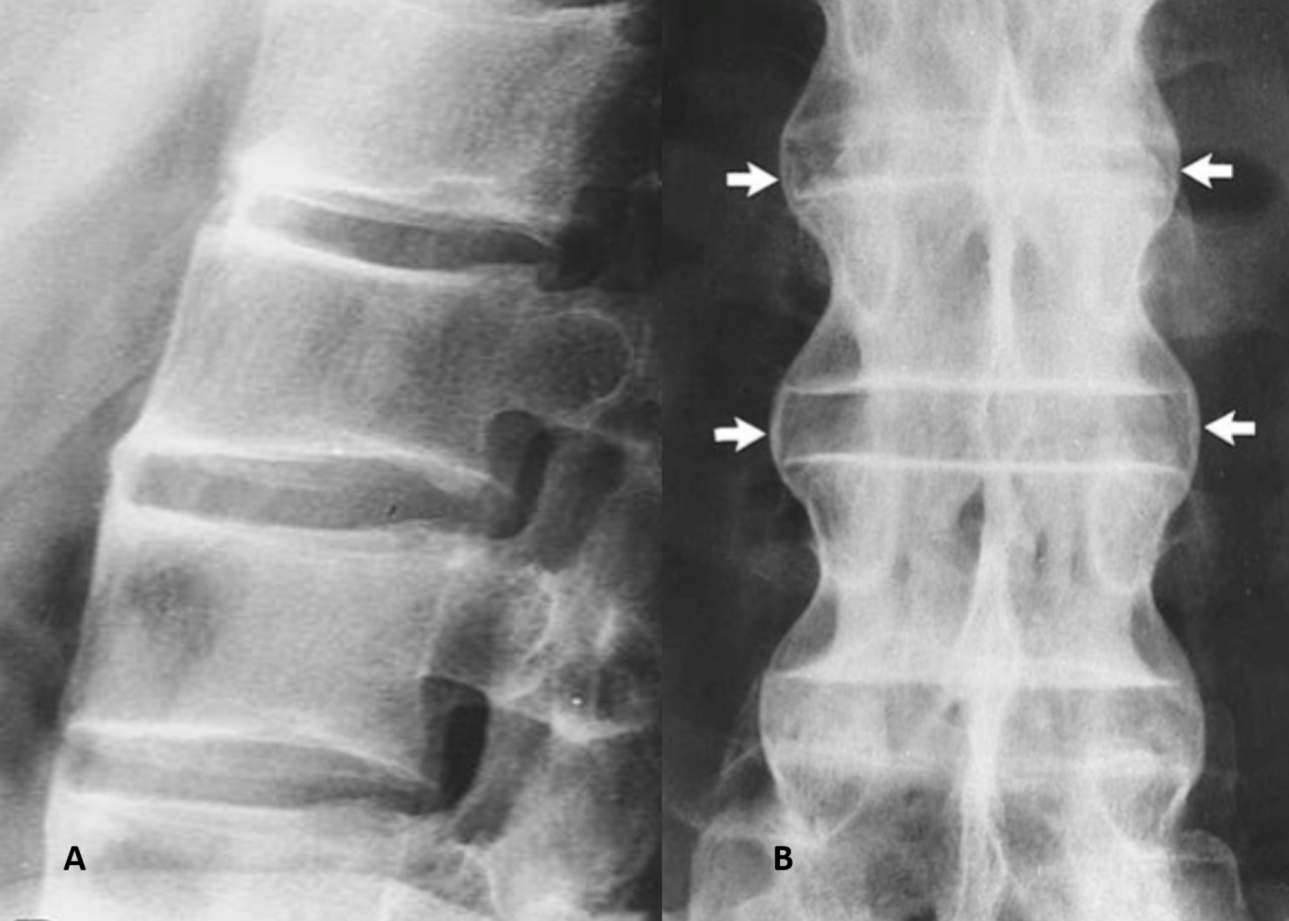
X-ray bamboo spine

Ankylosing spondylitis is a chronic seronegative systemic autoimmune condition, characterised by HLA-B27 complex, which affects the axial spine, and can cause uveitis, iritis, heart disease, pulmonary fibrosis, and aortic abnormalities. It causes stiffness, pain in the back, and makes the patient prone to spine fractures (Figures 4-5).

Fish vertebrae

**Figure 6:**
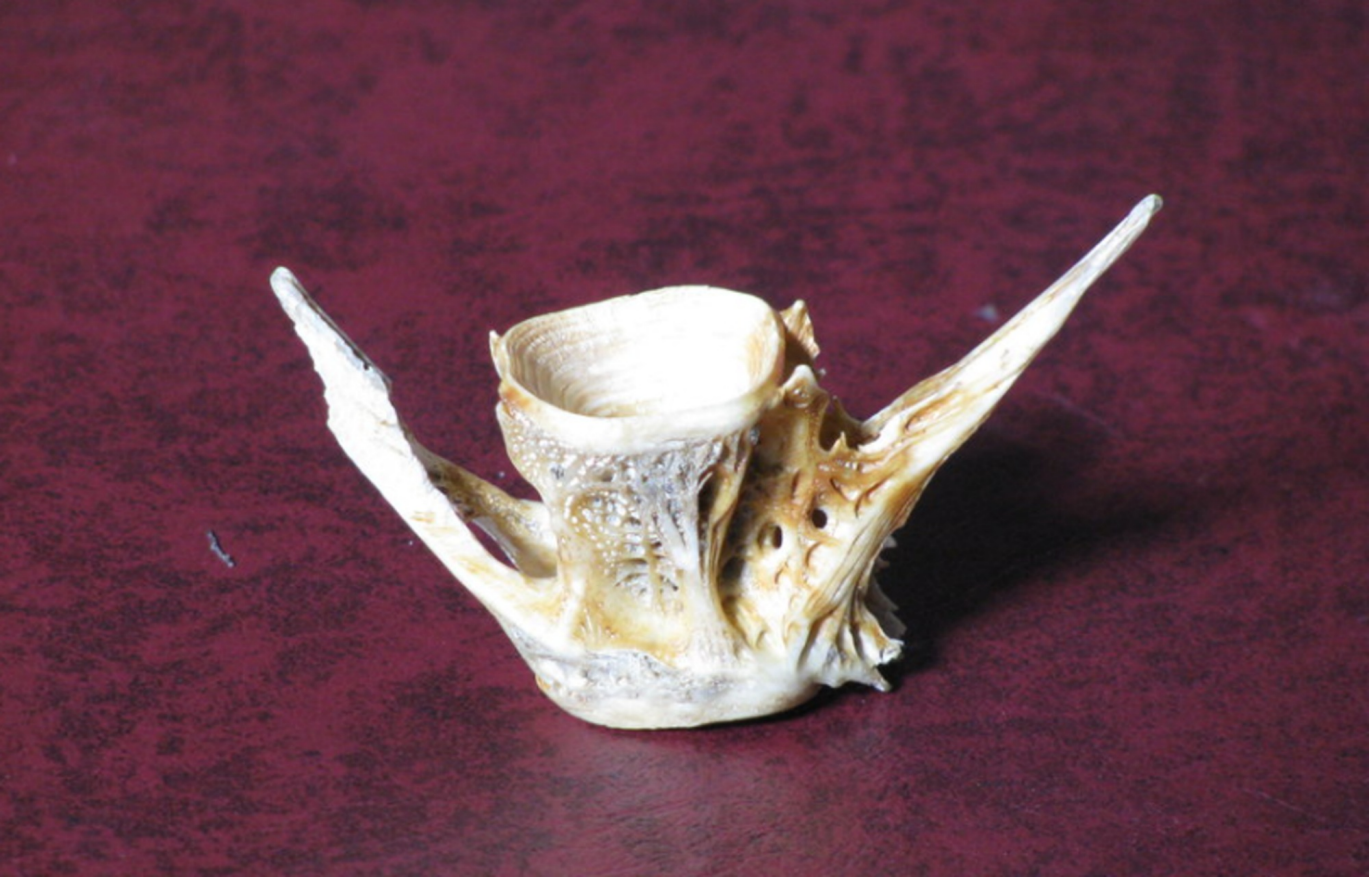
Vertebrae of a fish

**Figure 7:**
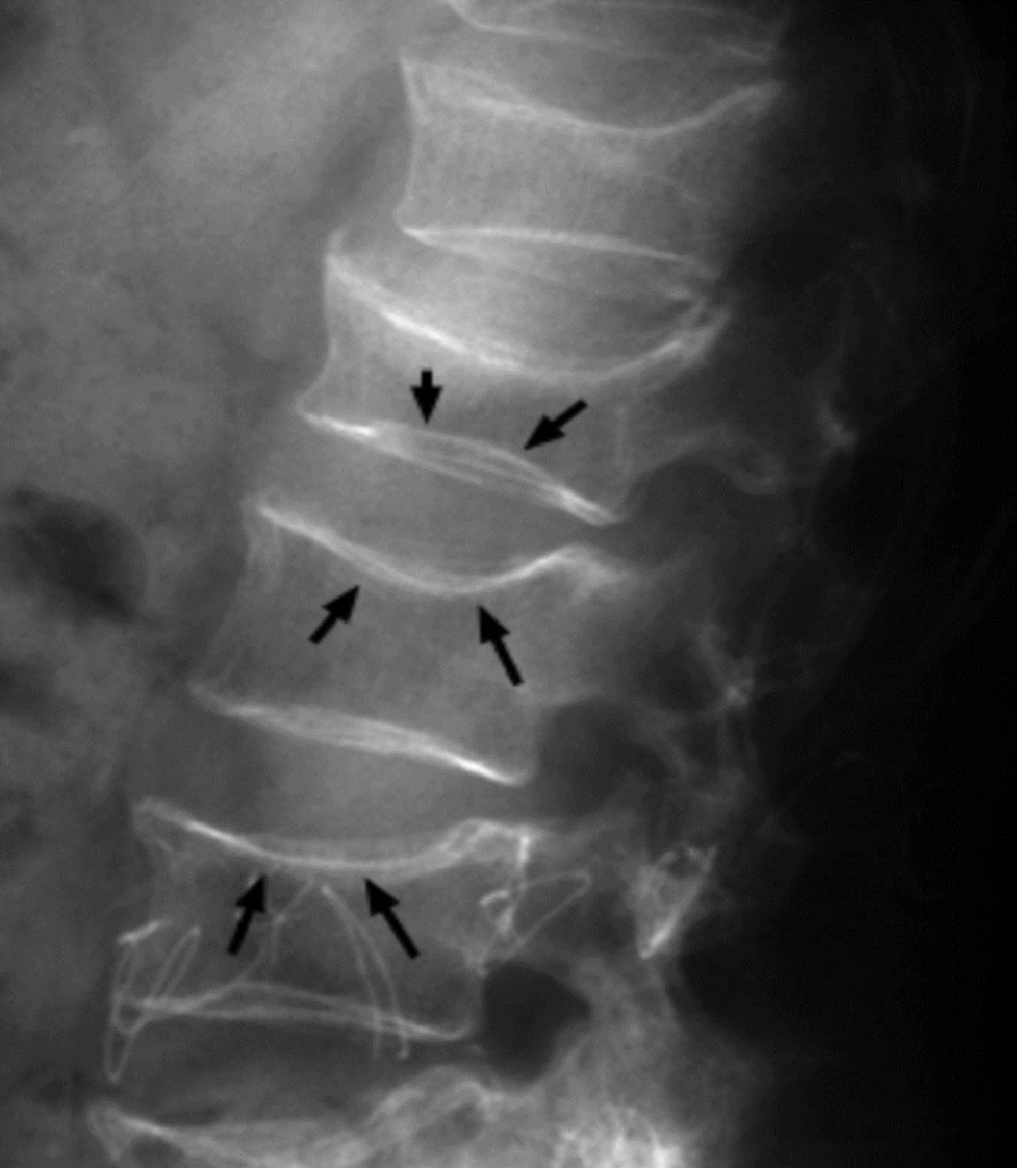
Fish vertebrae sign Biconcave vertebral body resembling fish vertebrae.

Fish vertebrae sign is the biconcave deformity of the vertebrae seen in osteopenia. The actual vertebrae of fish have depressions on their superior and inferior surfaces [4]. Clinically, fish vertebrae are seen in osteopenia or osteoporosis (Figures 6-7).

Ivory vertebrae

Ivory vertebrae is a sign denoting diffuse and uniform increases in vertebral opacity. There is no effect on the vertebral size or its adjacent intervertebral discs. Ivory vertebrae result from infectious or metastatic disorders. The pathophysiology of ivory vertebrae is when an infectious organism gets to the vertebrae and causes an inflammatory process resulting in inflammatory cells, vascular congestion, oedema, and thrombosis. Due to the compromised blood supply to the bone marrow and periosteum, dead bone forms and appears whiter and higher in opacity on x-rays than healthy bone tissue, hence, the “ivory” appearance [5].

**Figure 8:**
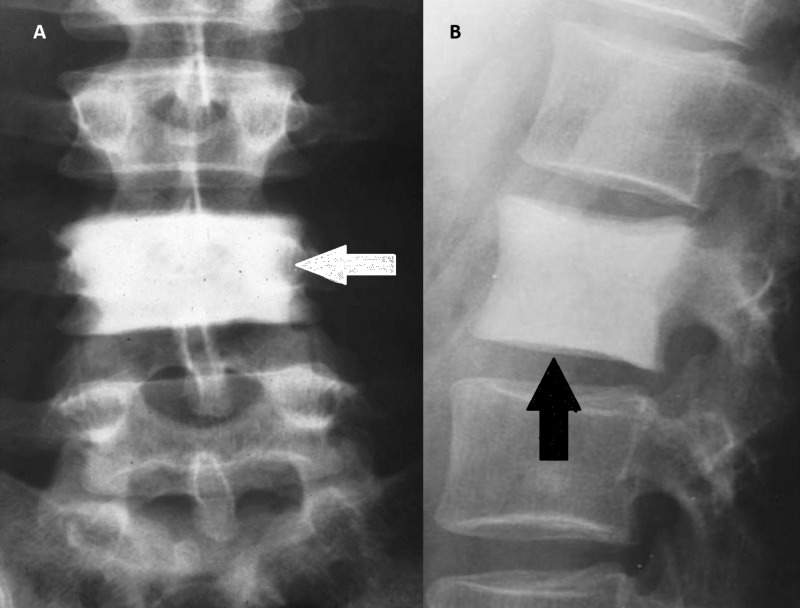
Ivory vertebrae Arrows showing diffuse homogenous ivory vertebrae.

Ivory vertebrae is a radiographic sign associated with many conditions including lymphomas, breast cancer, prostate cancer, Paget’s disease, and osteomyelitis (Figure 8).

Winking owl

Winking owl is the name of the sign when a pedicle is not visible on anteroposterior (AP) plane x-ray of the spine. On an AP spine x-ray, the two pedicles at each vertebral level look like an owl’s eyes. There is a shadow over the pedicle which makes it difficult to visualise the pedicle. The pathophysiology is from a tumour that has spread to the vertebral body that then spreads to the surrounding structures. It is also caused by spinal metastasis, tuberculosis, lymphoma or infections [6].

**Figure 9:**
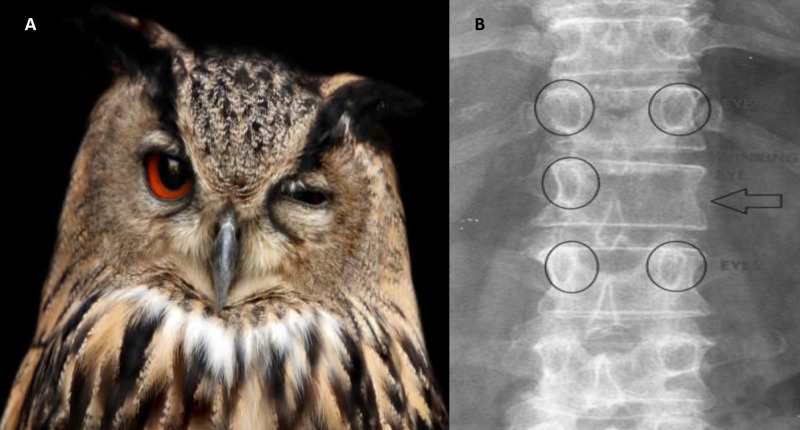
Winking owl sign A winking owl and x-ray showing similar appearance.

Looking at the winked owl appearance, the pedicle is the open eye, the pedicle which cannot be seen is the closed eye, and the spinous process looks like an owl’s beak (Figure 9).

Snake eye appearance

This is a magnetic resonance image (MRI) finding usually seen in cases of cervical myelopathy. The snake eye sign is seen in both T1 and T2 phase images of the cervical spine, and it is visible on axial plane MRI where the symmetric bilateral anterior horn grey matter mimics a pair of snake’s eyes [7].

**Figure 10:**
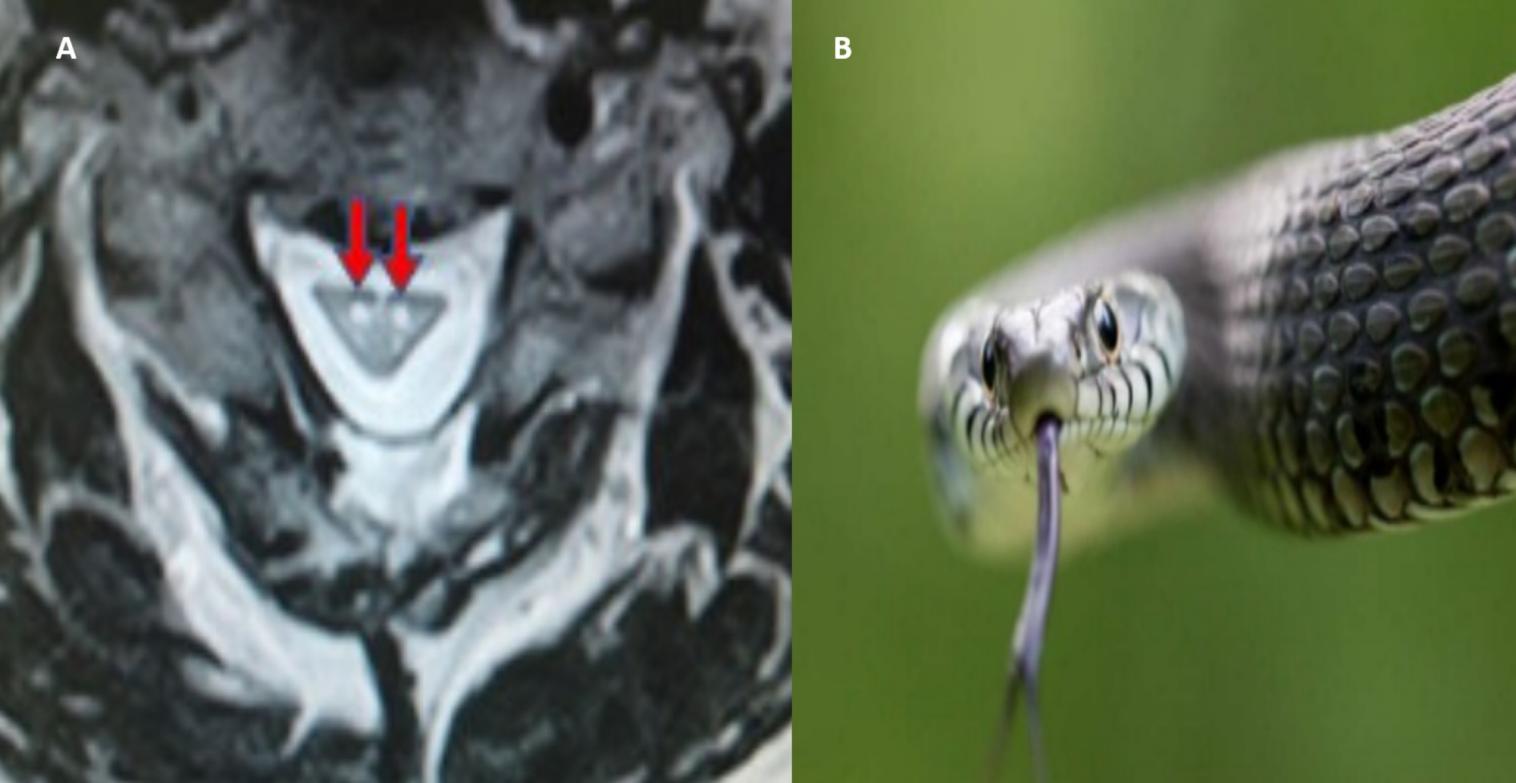
Snake eye sign Snake and red arrows showing spinal cord appearance resembling snake eyes.

It is seen in cervical myopathy and amyotrophic lateral sclerosis. Cervical myopathy is a clinical syndrome characterised by spinal cord compression, hand clumsiness, and gait imbalance (Figure 10).

Scalloping vertebrae

In healthy vertebrae, a small degree of concavity is present in the posterior vertebral body. In certain conditions, there is increased concavity of the posterior vertebral body, which is evident on lateral x-ray images of the spine. Scalloping vertebrae can also be visible in sagittal plane computed tomography (CT) scans and MRIs. Increased intraspinal pressure due to a mass can cause exaggerated concavity [8].

**Figure 11:**
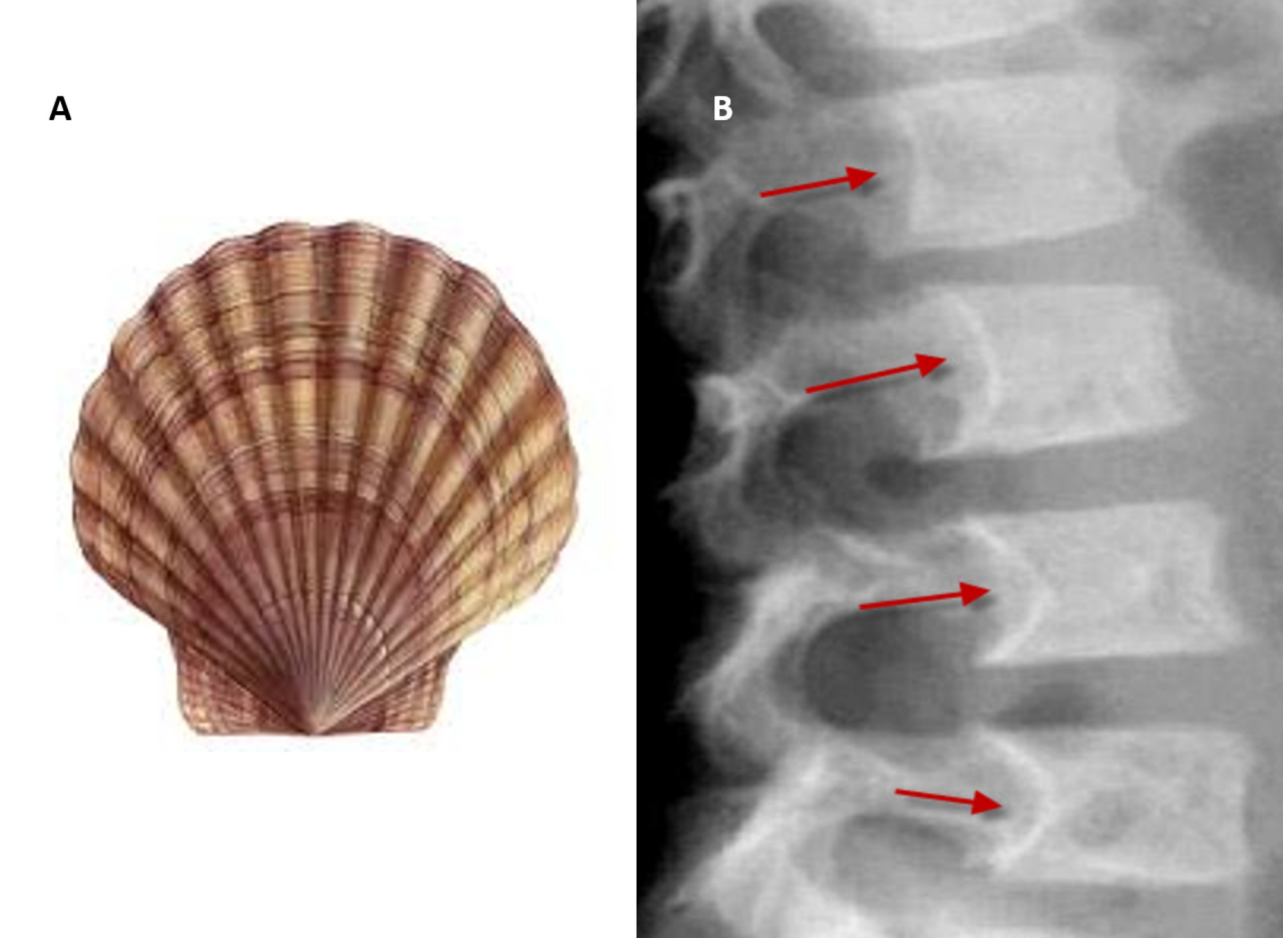
Scalloping vertebrae sign Red arrows showing scalloping of posterior vertebral body.

This sign is associated with intraspinal mass like spinal astrocytoma, ependymoma, schwannoma, neurofibroma, and achondroplasia, among others (Figure 11).

## Conclusions

This review illustrates the influence of natural phenomena in the naming of radiological findings in the spine. It is interesting to note the spine radiology signs inspired by nature, as they help to memorise the difficult concepts and are very helpful in explaining the findings to laymen for their better understanding since they cannot understand the difficult medical terms.
